# 

**Published:** 1890-07

**Authors:** 


### CONTENTS.

### *EDITORIALS.*

| | PAGE |
|---|---|
| The Use of Crude Drugs by Professed Homœopaths, . . . | 289 |
| The Repetition of the Dose, . . . . . . . . . . | 291 |
| Dr. Guernsey's Answer, . . . . . . . . . . . | 293 |

### *MISCELLANEOUS.*

| | |
|---|---|
| The Sycosis of Hahnemann. A. McNeil, M. D., . . . . . | 294 |
| British Medicinal Plants. Alfred Heath, M. D., F. L. S., . . | 295 |
| Obstetrics. Wm. Steinrauf, M. D., . . . . . . . . | 299 |
| Fatal Errors. J. H. Jackson, M. D., . . . . . . . . | 301 |
| Notes from Past Meetings of The Lippe Society, . . . . . | 304 |
| Reports of Clinical Cases. G. M. Pease, M. D., . . . . | 307 |
| Dysmenorrhœa. L. L. Helt, M. D., . . . . . . . . | 309 |
| Another Answer to Dr. Allen's Protest. G. W. Sherbino, M. D., . | 310 |
| Jarring, . . . . . . . . . . . . . . . | 312 |
| Epidemiological. Dr. Kunkel. Translated by S. L., . . . | 313 |
| The Value of Trivial Symptoms. G. M. Pease, M. D., . . . | 315 |
| La Grippe. Dr. Kunkel. Translated by S. L., . . . . . | 316 |
| Provings of Syphilinum. Samuel Swan, M. D., . . . . . | 318 |
| Provings. E. W. Berridge, M. D., . . . . . . . . | 320 |
| Correspondence. W. A. Yingling, M. D., . . . . . . . | 321 |
| A Study in Materia Medica. J. T. Kent, M. D., . . . . . | 322 |
| Dr. Guernsey's Defense. Egbert Guernsey, M. D., . . . . | 323 |
| Diphtheria. Wm. Steinrauf, M. D., . . . . . . . . | 324 |
| The Indiana Institute of Homœopathy, . . . . . . . | 325 |

### *BOOK NOTICES.*

| | |
|---|---|
| The Homœopathic Treatment of Alcoholism. Dr. Gallavardin, . | 328 |
| Electricity in the Diseases of Women. G. Betton Massey, M. D., . | 329 |
| The Guiding Symptoms of our Materia Medica. C. Hering, M. D., . | 329 |

### *NOTES AND NOTICES.*

Actinomycosis—Removals—"An Ischiophagus"—The Electric Railway as a Sanitary Measure—Consumption not Contagious—Statistics of Farm, Homes, and Mortgages—Southern Homœopathic Medical College—An Appeal from the Census Bureau—The Value of Journals to the Medical Profession—The Bromides—A Census of Hallucinations—Dr. Wm. B. Clarke—Who was your Great-Grandfather—Oh-don't-ology—Fun for Doctors, . . . . . . . . . . . . . 330–336

